# A Case of Central Diabetes Insipidus in a Patient With a Pineal Mass Suspected to Be a Germinoma: A Case Report

**DOI:** 10.7759/cureus.46103

**Published:** 2023-09-28

**Authors:** Akbar Hussain, Mythili Kanthi Gudipati, Edilfavia Uy, Jonathan Piercy, Shyam Ganti

**Affiliations:** 1 Internal Medicine, Appalachian Regional Healthcare, Harlan, USA; 2 Diabetes and Endocrinology, Appalachian Regional Healthcare, Harlan, USA

**Keywords:** radiological, neurosurgical, prednisone, infundibuloneurohypophysitis (inh), central diabetes insipidus (cdi)

## Abstract

Central diabetes insipidus (CDI) is a rare condition characterized by excessive urination and thirst due to vasopressin deficiency. The underlying cause of CDI remains unknown in many cases. Tumors are a leading cause of CDI in young individuals, with germinoma being the most prevalent. We present a case of a 22-year-old male diagnosed with infundibuloneurohypophysitis (INH) of unknown etiology. His pituitary stalk thickening partially responded to high-dose prednisone treatment; however, one year after initial diagnosis, a new pineal region mass was noted on imaging. Further evaluation revealed the mass to be most likely a germinoma. This case emphasizes the importance of ongoing clinical and radiologic follow-up in idiopathic cases of CDI. The patient's symptoms improved with desmopressin, but the presence of the pineal mass necessitates further comprehensive neurosurgical evaluation.

## Introduction

Central diabetes insipidus (CDI) is characterized by increased urination and thirst due to vasopressin deficiency. It can be familial, idiopathic, or secondary to any condition affecting the hypothalamus and posterior pituitary. Extensive diagnostic evaluations are often conducted, but in about 40% of cases, the underlying cause remains unknown [1]. Epidemiologically, tumors are the leading cause of diabetes insipidus in infants and adolescents, among which germinomas are the most prevalent cause [2]. This tumor develops from germ cells migrating into the central nervous system during fetal life, with 40% of cases being suprasellar, but it may also extend to the cerebrospinal fluid. The initial appearance on a magnetic resonance imaging (MRI) scan may be somewhat restricted because of a thickened pituitary stalk. This thickening can occur for various reasons, including idiopathic (of unknown cause), inflammatory, neoplastic (related to tumors), and infiltrative conditions. Consequently, it often becomes necessary to perform a histopathologic analysis to definitively establish the diagnosis [3,4]. We present a case of a 22-year-old with central diabetes insipidus. Initial brain MRI revealed infundibuloneurohypophysitis (INH) of unknown origin. Treatment included desmopressin and prednisone, resulting in mild improvement in pituitary stalk thickening. However, subsequent imaging identified a new pineal mass. CSF fluid analysis confirmed germinoma. Collaborative efforts with neurosurgery, radiation oncology, and oncology were initiated. A biopsy of the pineal mass was deferred, and the patient was scheduled for chemotherapy and radiation therapy.

## Case presentation

We present a case of a 22-year-old male who presented to our clinic with a two-month history of increased urinary frequency and excessive thirst. The patient reported voiding approximately 10-15 times per day, including four times at night. Increased thirst and exhaustion were present along with this. In addition, the patient reported negative neurologic symptoms. He denies smoking, drinking, or illicit drug use, and he denies taking any medication. Physical examination findings were unremarkable.

The patient, initially presenting with normal vital signs, underwent a comprehensive assessment. Initial laboratory results indicated normal blood glucose levels (75 mg/dL), a glycated hemoglobin (HbA1c) of 4.9%, and a sodium level of 142 mmol/L. Furthermore, urinalysis revealed specific gravity below 1.005, along with negative findings for ketones, blood, leukocyte esterase, and nitrates. However, a significant finding was a 24-hour urine collection output of 16 liters, which raised concerns about fluid balance.

On further evaluation, the patient underwent the water deprivation test. The patient displayed signs of impaired fluid balance, including elevated urine output and a significant decrease in body weight. Subsequent administration of desmopressin led to a notable improvement in these parameters. Urine output substantially decreased, and the urine became more concentrated, as indicated by increased urine osmolality and specific gravity. The shift in these measurements suggested a favorable response to desmopressin therapy. These findings point toward a potential diagnosis of diabetes insipidus, where desmopressin effectively countered excessive urine output and improved urine concentration, providing valuable insights for further diagnostic evaluation, as shown in Table 1. 

**Table 1:** The patient's values before and after the administration of desmopressin during the water deprivation test

Parameter	Normal Reference Range	Values Before Desmopressin Administration	Value After Desmopressin Administration
Sodium (mEq/L)	135-145	144	147
Plasma Osmolality (mOsm/kg)	275-295	300	314
Urine Osmolality (mOsm/kg)	500-800	83	92
Urine Output (cc/hour)	0.5 - 1.5 mL/kg/hr (adults)	400-500	150
Weight Change (lbs)	Not Applicable	Decreased by 6 lbs	Not Applicable
Urine Specific Gravity	1.010 - 1.030	Not Applicable	1.020
Urine Osmolality (mOsm/kg)	500-800	Not Applicable	539
Plasma Osmolality (mOsm/kg)	275-295	Not Applicable	292

**Figure 1:**
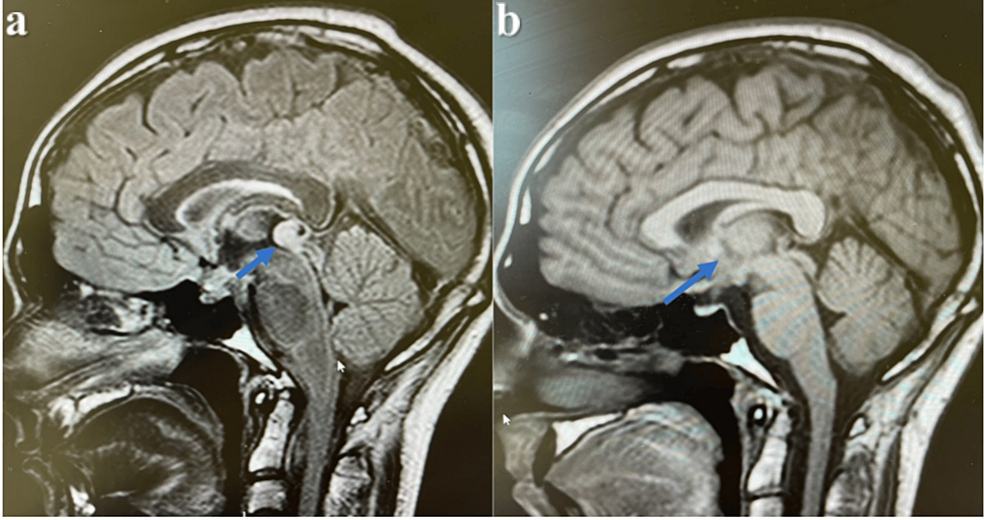
MRI brain diagnostic imaging reveals a prominent pineal region mass, and (b) Notable pituitary stalk thickening

Radiology consultation was sought due to specific magnetic resonance imaging (MRI) of the brain revealing an absent posterior pituitary bright spot and a thickened pituitary stalk measuring 4.2 mm, as shown in Figure 1a. The report suggested the possibility of an autoimmune or inflammatory disorder, specifically infundibuloneurohypophysitis, as the cause of central diabetes insipidus.

A comprehensive diagnostic process was undertaken, including evaluations of pituitary hormone levels and serological markers such as immunoglobulin G (IgG), alpha-fetoprotein (AFP), human chorionic gonadotropin (HCG), hepatitis A/B/C, quantiferon, and rapid plasma reagin (RPR). Additionally, computed tomography (CT) scans of the neck, chest, and abdomen, as well as a skeletal survey, were conducted to investigate potential causes of pituitary stalk thickening, as detailed in Table 2. The spectrum of possible underlying conditions considered encompassed autoimmune, inflammatory, infectious, and neoplastic origins. Importantly, conditions like IgG4-related disease and ANCA-negative granulomatous disorders were ruled out, as supported by negative test results for HIV, hepatitis A/B/C, QuantiFERON, and RPR. This thorough diagnostic approach aimed to pinpoint the precise etiology of the pituitary stalk thickening. Throughout various assessments, the functionality of the anterior pituitary hormones remained intact. The medical approach included seeking a neurosurgical consultation, although the decision was made to defer a biopsy and initiate empirical prednisone therapy.

**Table 2:** Hormone and test results over follow-up TSH: Thyroid-Stimulating Hormone, FT4: Free Thyroxine (T4), FSH: Follicle-Stimulating Hormone, LH: Luteinizing Hormone, IGF1: Insulin-Like Growth Factor 1, GH: Growth Hormone, ACTH: Adrenocorticotropic Hormone, IgG: Immunoglobulin G, IgG4: Immunoglobulin G4

Lab Tests	Reference Range	First	2 Months After	4 Months After	Most Recent Visit
TSH (ulU/mL)	0.4 - 4.0	2.139 (Normal)	Normal	Normal	Normal
FT4 (ng/dL)	0.9 - 1.7	1.06 (Normal)	Normal	Normal	Normal
FSH (mIU/mL)	Male (1.5 - 12.4)	4.37 (Normal)	Normal	Normal	43.3 (Elevated)
	Female (1.4 - 18.1)				
LH (mIU/mL)	Male (1.24 - 7.8)	6.0 (Normal)	Normal	Normal	30 (Elevated)
	Female (1.05 - 16.7)				
Testosterone (ng/dL)	Male (270 - 1070)	698 (Normal)	Normal	Normal	Normal
	Female (15 - 70)				
Free Testosterone (ng/dL)	Male (50 - 210)	0.0756 (Normal)	Normal	Normal	Normal
Prolactin (ng/mL)	4.0 - 15.2	11 (Slightly Elevated)	Slightly Elevated	Slightly Elevated	Slightly Elevated
IGF1 (ng/mL)	Age-dependent	142 (Normal)	Normal	Normal	Normal
ACTH (pg/mL)	7.2 - 63.3	16.9 (Normal)	43.3 (Elevated)	30 (Elevated)	Elevated
Cortisol (ug/dL)	Morning: 6.2 - 19.4	15.82 (Normal)	16 (Normal)	13 (Normal)	Decreased
IgG (mg/dL)	700 - 1600	631 (Low)	631 (Low)	Below Normal Range	Below Normal Range
IgG4 (mg/dL)	4 - 86	28 (Normal)	28 (Normal)	Within Normal Range	Within Normal Range

During the patient's follow-up, his symptoms improved significantly after starting desmopressin, and the dosage was adjusted based on their clinical response. The patient continued to attend regular appointments with neurosurgery specialists and underwent subsequent brain MRI assessments. About a year after the initial diagnosis, the MRI revealed that while the mild pituitary stalk thickening remained stable, a new pineal mass lesion emerged, measuring approximately 9mm in the anterior-posterior dimension, 9mm in the craniocaudal dimension, and 1.3cm in the transverse dimension. This enhanced mass was located near the upper part of the quadrigeminal plate, as illustrated in Figure 1b.

Following a lumbar puncture, the patient underwent cerebrospinal fluid (CSF) analysis and repeated biochemical testing, which indicated the presence of a germinoma. In response, a collaborative approach involving specialists in neurosurgery, radiation oncology, and oncology was adopted. As a result, a decision was made to postpone the biopsy of the pineal mass, and the patient is currently in the process of scheduling a treatment plan involving both chemotherapy and radiation therapy. It's worth noting that while the patient continues to show intracranial hypothalamic involvement (INH), the cause has transitioned from being idiopathic to being attributed to germinoma, offering a more precise and specific diagnosis.

Regular imaging studies will be performed to assess the tumor's size, location, and potential metastasis. Neurologic examinations will be conducted frequently to evaluate motor, sensory, and cranial nerve function and signs of increased intracranial pressure. Close monitoring of hCG biomarkers will be done to track treatment responses. Furthermore, healthcare providers will actively monitor the patient's anterior pituitary hormone function to detect any potential disruptions caused by the tumor or chemoradiation therapy. The collaborative medical team, including neuro-oncologists, neurosurgeons, endocrinologists, radiologists, oncology nurses, and psychologists, will work together to ensure a comprehensive and tailored plan for the patient. This approach will facilitate effective treatment strategies, promote patient well-being, and enable timely interventions as required.

## Discussion

Central diabetes insipidus (CDI) is a rare disorder characterized by polyuria and polydipsia resulting from inadequate secretion of vasopressin, also known as antidiuretic hormone (ADH), from the posterior pituitary gland. It can be classified as familial, idiopathic, or secondary, with up to 50% being of idiopathic origin [5,6]. In this case report, we present a unique scenario where a patient initially was diagnosed with idiopathic CDI and eventually was found to have a pineal region mass that was highly suspected to be a germinoma after cerebrospinal fluid (CSF) analysis.

The initial diagnostic challenge in our case was the presentation of CDI in a young adult with an unclear etiology. While it is well known that idiopathic CDI involves dysfunction of the hypothalamic-neurohypophysial system, which frequently manifests on imaging as a thickened pituitary stalk [7], the precise underlying cause is frequently elusive. Various potential etiologies of pituitary stalk thickening were considered, including autoimmune, inflammatory, infectious, and neoplastic origins. These possibilities were methodically investigated through comprehensive pituitary hormone testing, serological assessments, and radiological studies. While conditions like IgG4-related disease and anti-neutrophil cytoplasmic antibodies (ANCA) negative granulomatous disorders were ruled out with supporting negative results in specific tests, the absence of a lesion was not due to oversight; rather, it was not initially present, and its eventual manifestation occurred due to the patient's early identification of its course. 

As seen in our patient, the clinical presentation of increased urination and thirst prompted an extensive diagnostic evaluation, including laboratory tests and imaging studies. Notably, the initial response to desmopressin and partial improvement with prednisone raised suspicion of an autoimmune or inflammatory process. However, the subsequent identification of a pineal region mass on repeat MRI complicated the diagnostic trajectory. The distinction between various causes of pituitary stalk thickening is pivotal in guiding appropriate management. Imaging results can be similar for both inflammatory conditions like lymphocytic infundibula-hypophysitis and tumors like germinomas.

Germinomas are uncommon, with a reported incidence of 0.1 per 100,000 people in the United States. Germinoma, a germ cell tumor originating from fetal cells migrating into the central nervous system, is a common cause of intracranial tumors leading to CDI [2]. They comprise about 0.5%-3% of all primary central nervous system tumors, with a male preponderance. They occur more commonly in children and adolescents and are generally rare in adults. The location, size, degree of involvement, and type of endocrine dysfunction all play a significant role in the signs and symptoms. This radiosensitive tumor is associated with a favorable prognosis, with a five-year survival rate of 85% [8].

Accurate diagnosis is crucial, as it dictates treatment strategies and prognosis. Histopathological analysis, often achieved through biopsy, remains the gold standard for definitive diagnosis in cases where imaging alone is inconclusive. In our patient, the CSF analysis showing elevated beta HCG tumor markers was deemed confirmatory for germinoma. On imaging, germinomas usually show up as well-defined masses, and they tend to form in the pineal and suprasellar regions.

When diabetes insipidus first manifests, it is crucial to rule out the potential of a tumor by having an MRI every three to six months (depending on how quickly the pituitary stalk thickens) [9]. After a three-year follow-up, there is a very low chance that a tumor lesion would still exist; therefore, two further years of yearly MRIs are possible [10]. Clinical and hormonal monitoring is imperative [11].

The management of CDI associated with a suspected germinoma involves a multidimensional approach. Prompt referral to a neurosurgery specialist, as demonstrated in our case, is essential for evaluating the extent of the mass, determining the need for surgical intervention, and planning subsequent treatment modalities. Germinomas are sensitive to radiation therapy, and chemotherapy can also play a role, making an accurate diagnosis a critical factor in guiding therapeutic decisions. The absence of the lesion was not due to oversight; it was not initially present, and its eventual manifestation occurred because the patient was identified early in its course.

## Conclusions

Central diabetes insipidus (CDI) poses complex diagnostic challenges. This case emphasizes the need for persistent evaluation and monitoring, as underlying causes may emerge over time. Initially diagnosed as idiopathic, the CDI was later complicated by the discovery of a pineal region mass, confirmed as a germinoma. Timely multidisciplinary collaboration and accurate diagnosis are crucial for effective management, given the germinoma's responsiveness to radiation and chemotherapy. Early detection and patient-centered care are key to optimizing outcomes in such intricate CDI cases associated with intracranial tumors.
